# Robustness of VMAT to setup errors in postmastectomy radiotherapy of left-sided breast cancer: Impact of bolus thickness

**DOI:** 10.1371/journal.pone.0280456

**Published:** 2023-01-24

**Authors:** Yipeng He, Sijia Chen, Xiang Gao, Lirong Fu, Zheng Kang, Jun Liu, Liwan Shi, Yimin Li

**Affiliations:** Department of Radiation Oncology, The First Affiliated Hospital of Xiamen University, Xiamen, China; Acibadem Mehmet Ali Aydinlar University, School of Medicine / Acibadem Mehmet Ali Aydinlar University, Research Institute of Senology, TURKEY

## Abstract

**Background:**

Volumetric modulated arc therapy (VMAT) with varied bolus thicknesses has been employed in postmastectomy radiotherapy (PMRT) of breast cancer to improve superficial target coverage. However, impact of bolus thickness on plan robustness remains unclear.

**Methods:**

The study enrolled ten patients with left-sided breast cancer who received radiotherapy using VMAT with 5 mm and 10 mm bolus (VMAT-5B and VMAT-10B). Inter-fractional setup errors were simulated by introducing a 3 mm shift to isocenter of the original plans in the anterior-posterior, left-right, and inferior-superior directions. The plans (perturbed plans) were recalculated without changing other parameters. Dose volume histograms (DVH) were collected for plan evaluation. Absolute dose differences in DVH endpoints for the clinical target volume (CTV), heart, and left lung between the perturbed plans and the original ones were used for robustness analysis.

**Results:**

VMAT-10B showed better target coverage, while VMAT-5B was superior in organs-at-risk (OARs) sparing. As expected, small setup errors of 3 mm could induce dose fluctuations in CTV and OARs. The differences in CTV were small in VMAT-5B, with a maximum difference of -1.05 Gy for the posterior shifts. For VMAT-10B, isocenter shifts in the posterior and right directions significantly decreased CTV coverage. The differences were -1.69 Gy, -1.48 Gy and -1.99 Gy, -1.69 Gy for ΔD_95%_ and ΔD_98%_, respectively. Regarding the OARs, only isocenter shifts in the posterior, right, and inferior directions increased dose to the left lung and the heart. Differences in VMAT-10B were milder than those in VMAT-5B. Specifically, mean heart dose were increased by 0.42 Gy (range 0.10 ~ 0.95 Gy) and 0.20 Gy (range -0.11 ~ 0.72 Gy), and mean dose for the left lung were increased by 1.02 Gy (range 0.79 ~ 1.18 Gy) and 0.68 Gy (range 0.47 ~ 0.84 Gy) in VMAT-5B and VMAT-10B, respectively. High-dose volumes in the organs were increased by approximate 0 ~ 2 and 1 ~ 3 percentage points, respectively. Nevertheless, most of the dosimetric parameters in the perturbed plans were still clinically acceptable.

**Conclusions:**

VMAT-5B appears to be more robust to 3 mm setup errors than VMAT-10B. VMAT-5B also resulted in better OARs sparing with acceptable target coverage and dose homogeneity. Therefore 5 mm bolus is recommended for PMRT of left-sided breast cancer using VMAT.

## Introduction

Female breast cancer has taken the place of lung cancer as the most common cancer in the world, with an estimated 2.3 million patients diagnosed with this disease in 2020 [1]. Though most of the breast cancer patients in the United States choose breast conserving surgery [2], modified radical mastectomy remains the most common technique for patients in China [3]. Adjuvant radiotherapy has been recommended for breast cancer patients treated with mastectomy for the benefits of improving locoregional recurrence rates and reducing cancer-related mortality [4–6].

Tangential field based three-dimensional conformal radiotherapy (3DCRT) is the standard technique for treatment planning of breast cancer. For postmastectomy radiotherapy (PMRT) in patients (especially those with left-sided breast cancer) with concave chest and/or regional lymph nodes, it is challenging for the traditional 3DCRT to deliver optimal target coverage and acceptable dose to the adjacent organs. To address this issue, advanced techniques including intensity-modulated radiation therapy (IMRT) and volumetric modulated arc therapy (VMAT) have been introduced to PMRT of breast cancer [7, 8]. Compared with 3DCRT, IMRT and VMAT showed better target coverage, dose homogeneity and conformity, and lower dose to the heart and left lung [9, 10]. The improved dose homogeneity in the target and intermediate-high dose to the organs was reported to be associated with lower skin and lung toxicity [11, 12]. Furthermore, VMAT was more efficient than IMRT in terms of monitor units and treatment time [13]. Shorter treatment time is beneficial for reducing the possibility of dose uncertainty caused by intra-fractional patient movement.

Inter-fractional patient setup errors are another source of dose variability. Generally, the errors are in the order of several millimeters when pretreatment setup verifications have been routinely performed [14–16]. Considering the high modulations and steep dose fall-off in VMAT, a tiny setup error of several millimeters is capable to induce significant dose loss in the target [17–20]. Liao et al. found that a 3 mm setup error appeared to deteriorate plan quality of VMAT for locally advanced breast cancer [19]. In another paper, similar results were recorded in VMAT with 5 mm setup errors in early-stage breast cancer patients [20]. Underdosage in the target caused by patient setup uncertainty was related to the local failures in head and neck cancer treated with VMAT [18], suggesting the importance of plan robustness. Plan robustness refers to sensitivity of planned dose to uncertainties, such as inter-fractional setup errors. Recently, plan robustness was proposed to be included when evaluating plan quality [21].

Bolus is a common tool in breast PMRT, which has been used in compensating the intrinsic build-up effect of megavolt photons. For VMAT plans, bolus also plays a role in achieving skin flash, namely, extending field fluences out into the air to account for the respiratory motions and/or tissue deformations. A guideline developed by the American College of Radiology (ACR) recommended utilizing bolus for photons with 6MV or higher energy [22]. However, bolus thickness remains heterogeneous in clinical practice, typically ranging from 2 mm to 10 mm [23, 24]. Massive variations in bolus thickness in the clinic may not only bring difficulties in producing consistent plan quality, but also increase the probability of treatment errors. In a phantom-based dosimetric study, Lobb demonstrated that bolus thickness had an effect on plan robustness of IMRT when mimicking scalp cancer irradiation with tomotherapy [25]. However, reports on such effects in breast cancer with VMAT are scarce.

The main purpose of this study is to investigate impact of bolus thickness on robustness of VMAT against setup errors in PMRT of left-sided breast cancer. Dose distribution in the targets and the OARs in VMAT with different bolus thicknesses are also evaluated since most of the published reports only focused on skin dose [26, 27].

## Materials and methods

### Patients and volumes delineation

Ten consecutive patients diagnosed with left-sided breast cancer and treated with mastectomy were randomly selected and enrolled in this retrospective study. The mean age was 54±9 years (range 36–67). The patients received PMRT in our department between January and June 2020 using VMAT and daily bolus. All the patients completed the treatment course without interruption or early cessation. This study was reviewed and approved by the Institutional Review Boards of the First Affiliated Hospital of Xiamen University. Written informed consent for the patients was waived since the plans in this study were research use only, and patient information was fully anonymized.

The patients were immobilized in supine position and free breath with hands above their heads using a breast board with head holder (CVICO Medical Solutions, Coralville, USA). Computed tomography (CT) images were obtained using a 16-slice CT scanner (GE Healthcare, Chicago, USA) with 5 mm slice thickness. The acquired images were subsequently transferred into the Eclipse treatment planning system version 11 (Varian Medical Systems, Palo Alto, USA).

An experienced radiation oncologist contoured the clinical target volume (CTV) including the chest wall (CW) and lymph nodes around the supraclavicular fossa (SCF). A uniform 5 mm margin was added to the CTV to form the planning target volume (PTV). The PTV was restricted at least 2 mm (2 mm for the target around CW and 5 mm for the nodes around SCF) from the skin surface. For plan evaluation, the CTV was confined to the edge of the PTV. The lungs, heart, and contralateral breast were delineated as OARs using an automatic contouring tool developed by Manteia (Manteia, Xiamen, China). The radiation oncologist would revise these OARs if necessary.

### Treatment planning

**Fig 1:**
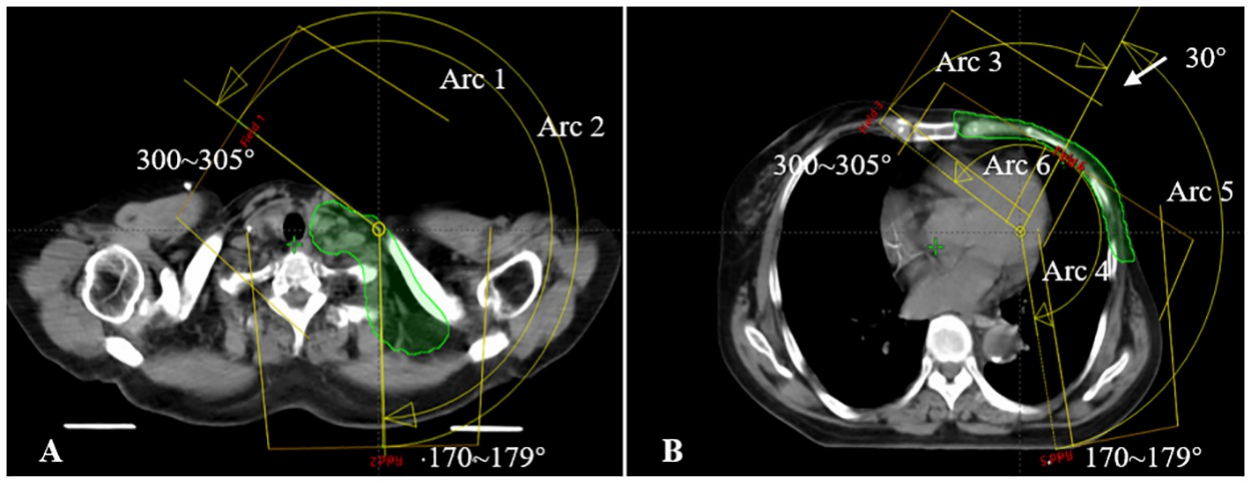
Schematic diagram showing the planning target volume (PTV) and beam arrangements for a representative patient. A, the two continuous ~240° arcs that start at 300 ~ 305° and stop at 170 ~ 179° for lymph nodes in SCF; B, the four split sub-arcs obtained by splitting two ~240° arcs at 30° for the chest wall.

All plans were generated in Eclipse for a Unique linear accelerator (Varian Medical Systems, Palo Alto, USA) with Millennium 120 multileaf collimator (MLC). Beam arrangements are presented in Fig 1: two continuous ~ 240° arcs (Arcs 1 and 2) with 6MV photons were used to irradiate the lymph nodes in SCF and four split arcs (Arcs 3–6) with the same energy for the CW. The arrangements of the split arcs were similar to that of the four-arc VMAT described by Lai et al. [28]. The collimator angles were set to 2 ~ 15° and 347 ~ 358° to minimize irradiation dose to the adjacent organs. The width of X jaw was limited to 18 cm for Arcs 1 ~ 2 and <14 cm for Arcs 3 ~ 6. Plans were optimized using the progressive resolution optimizer algorithm (PRO). Final dose calculation was conducted using the anisotropic analytical algorithm (AAA) with 2.5 mm grid size.

For VMAT-10B, extended PTV and 10 mm bolus illustrated in ref 28 were used for plan optimization. The prescription dose (PD) was 50 Gy with daily 2 Gy over 5 weeks. Plans were normalized to achieve at least 95% of PTV covered by PD and at least 99% of PTV covered by 95% of PD, meanwhile keeping the hot spot, defined as 110% of PD, as low as possible. The objectives for the OARs were as follows: mean dose (D_mean_) < 5~6 Gy and V_20Gy_ < 10% (V_xGy_: volume of organs receiving minimum dose of x Gy) for the heart; V_5Gy_ < 60%, V_20Gy_ < 30% and D_mean_ <15 Gy for the left lung; D_mean_ < 5 Gy for the contralateral lung and breast and keeping the V_5Gy_ as low as achievable. VMAT-5B was recalculated from VMAT-10B by replacing the 10 mm bolus with a 5 mm one without changing other parameters.

Inter-fractional setup errors were simulated by introducing 3 mm shifts to the isocenter of VMAT-5B and VMAT-10B in anterior-posterior, superior-inferior, and left-right directions. Afterwards, the plans were recalculated on the basis of the original planning CT without changing other parameters. For each patient, a total of twelve perturbed plans were generated.

### Plan evaluation

Dose volume histograms (DVHs) were collected and dosimetric parameters were extracted for plan evaluation. For both PTV and CTV, V_95%_, V_110%_ (volume receiving 95% and 110% of PD, respectively), D_95%_, D_98%_, D_2%_ (minimum dose in 95%, 98% and 2% of target, respectively) and D_mean_ were measured. Homogeneity index (HI) was calculated as HI = (D_2%_−D_98%_)/D_50%_. Plans with HI close to zero were considered with homogeneous dose distribution in the target. For OARs, V_xGy_, D_mean_ and maximum dose, defined as a minimum dose to 1 cm^3^ of the OARs (D_1cc_), were recorded.

Plan robustness between VMAT-5B and VMAT-10B was compared using the absolute differences between the perturbed plans and the original ones (Δdose = D_perturbed_−D_original_) in CTV, the left lung, and the heart. Specifically, ΔD_95%_, ΔD_98%_, ΔD_2%_, ΔV_110%_ for CTV; ΔV_20Gy_, ΔV_30Gy_, ΔV_40Gy_, ΔD_mean_ for the left lung, and ΔV_20Gy_, ΔV_30Gy_, ΔD_mean_ for the heart were evaluated.

### Statistics analysis

Quantitative data were presented as mean and standard deviations (SD). Normality of all the data was evaluated using the Kolmogorov–Smirnov test. Two-tailed t-test and paired Wilcoxon signed-rank test was utilized to compare the difference between VMAT-5B and VMAT-10B for normally distributed and non-normally distributed data, respectively using a significant level of 0.05. Statistical analyses were completed with R Version 4.0.5 (Foundation for Statistical Computing, Vienna, Austria) and the R Studio software.

## Results

### Dose distribution in VMAT-5B and VMAT-10B

**Fig 2:**
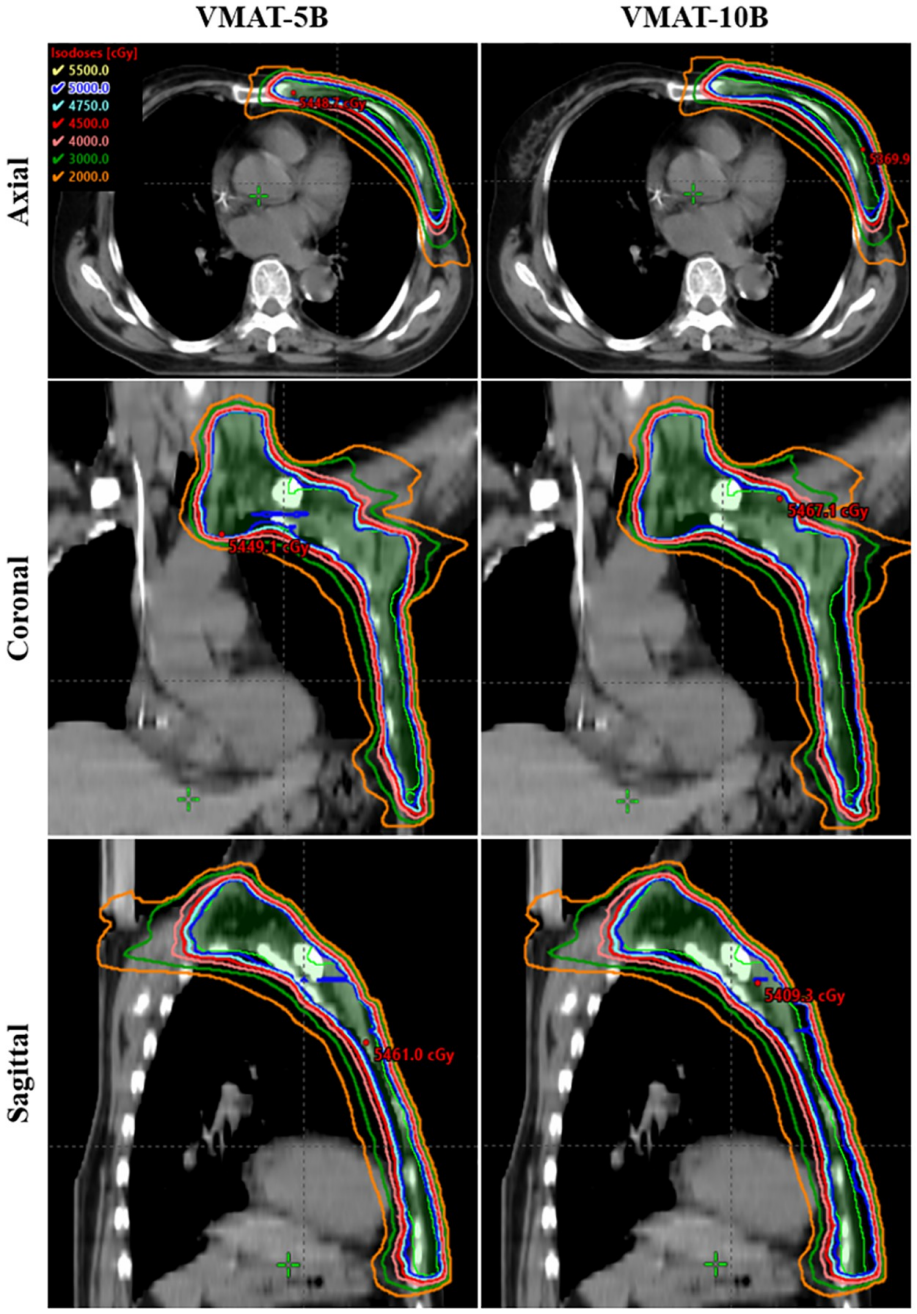
Dose distribution in the axial (upper panels), coronal (middle panels) and sagittal (lower panels) planes for a typical patient with VMAT-5B (left panels) and VMAT-10B (right panels), respectively.

Fig 2 shows dose distribution in axial, coronal, and sagittal planes of a representative patient with VMAT-5B and VMAT-10B. No significant differences were observed except the well extended isodose lines along the anterior chest wall in VMAT-10B. Dosimetric parameters of PTV and CTV are summarized in Table 1. Both VMAT-5B and VMAT-10B resulted in great target coverage with V_95%_ > 99.5%, D_95%_ ≈ 50.0 Gy and minimum dose (D_98%_) ≥ 49.0 Gy. Nevertheless, VMAT-10B had better dose coverage in CTV and homogeneity in PTV and CTV, compared with VMAT-5B.

**Table 1:** Dosimetric comparison between VMT-5B and VMAT-10B for the targets.

Parameters	VMAT-5B	VMAT-10B	*P* value
PTV			
V_95%_ (%)	99.59 ± 0.33	99.58 ± 0.34	0.799
D_95%_ (Gy)	49.9 ± 0.22	50.07 ± 0.10	0.074
D_98%_ (Gy)	49.0 ± 0.35	49.00 ± 0.32	0.445
D_mean_ (Gy)	52.10 ± 0.28	52.13 ± 0.36	0.878
D_2%_ (Gy)	54.20 ± 0.43	53.78 ± 0.46	**<0.001**
V_110%_ (%)	0.39 ± 0.59	0.06 ± 0.12	**0.005** *
HI	0.10 ± 0.01	0.09 ± 0.01	**<0.001**
CTV			
V_95%_ (%)	99.90 ± 0.04	99.98 ± 0.03	**0.028** *
D_95%_ (Gy)	50.70 ± 0.35	51.23 ± 0.35	**<0.001**
D_98%_ (Gy)	50.30 ± 0.37	50.87 ± 0.35	**<0.001**
D_mean_ (Gy)	52.30 ± 0.31	52.43 ± 0.41	0.391
D_2%_ (Gy)	54.30 ± 0.45	53.80 ± 0.46	**<0.001**
V_110%_ (%)	0.47 ± 0.66	0.04 ± 0.08	**0.005** *
HI	0.08 ± 0.01	0.06 ± 0.01	**<0.001**

The values in bold indicate significant differences.

*Significant differences with paired Wilcoxon signed-rank test.

Table 2 summarizes dosimetric parameters for the OARs. All the optimization criteria for the OARs mentioned in the ‘Materials and methods’ section were met for VMAT-5B and VMAT-10B. Dose to the heart and both lungs were significantly improved in VMAT-5B. However, for the right breast, the V_5Gy_ and D_1cc_ were slightly increased in VMAT-5B (20.14% vs 19.84% for V_5Gy_, *P* = 0.011; 14.29 Gy vs 13.94 Gy for D_1cc_, *P* <0.001), while D_mean_ was comparable between the two groups.

**Table 2:** Dosimetric comparison between VMAT-5B and VMAT-10B for the OARs.

Parameters	VMAT-5B	VMAT-10B	*P* value
Left lung			
V_5Gy_ (%)	51.16 ± 3.61	53.17 ± 3.82	**<0.001**
V_20Gy_ (%)	23.11 ± 2.26	23.67 ± 2.33	**<0.001**
V_30Gy_ (%)	15.21 ± 1.54	15.46 ± 1.54	**<0.001**
V_40Gy_ (%)	8.67 ± 1.32	8.71 ± 1.34	0.445
D_mean_ (Gy)	12.53 ± 0.91	12.80 ± 0.93	**<0.001**
Heart			
V_5Gy_ (%)	22.01 ± 4.50	24.59 ± 4.07	**<0.001**
V_20Gy_ (%)	3.20 ± 1.49	3.35 ± 1.50	**<0.001**
V_30Gy_ (%)	1.17 ± 0.89	1.21 ± 0.90	**<0.001**
D_mean_ (Gy)	4.43 ± 0.53	4.71 ± 0.50	**<0.001**
D_1cc_ (Gy)	38.03 ± 5.99	38.03 ± 5.66	0.880
Right lung			
V_5Gy_ (%)	22.54 ± 6.27	23.22 ± 6.21	**<0.001**
V_20Gy_ (%)	0.30 ± 0.40	0.30 ± 0.37	0.859
D_mean_ (Gy)	3.84 ± 0.54	3.90 ± 0.53	**0.001**
Right breast			
V_5Gy_ (%)	20.14 ± 5.76	19.84 ± 5.57	**0.011**
D_mean_ (Gy)	3.88 ± 0.47	3.86 ± 0.45	0.076
D_1cc_ (Gy)	14.29 ± 2.60	13.94 ± 2.49	**<0.001**

The values in bold indicate significant differences.

### Plan robustness

Table 3 presents the mean ΔD_95%_, ΔD_98%_, ΔD_2%_, and ΔV_110%_ for CTV in VMAT-5B and VMAT-10B. The setup errors resulted in insufficient target coverage in VMAT-10B, regardless of shifting directions. The ΔD_95%_ and ΔD_98%_ were of -1.69 ~ -0.25 Gy and -1.99 ~ -0.23 Gy, respectively. Maximum reduction was observed in the posterior direction (-1.69 and -1.99 Gy), followed by the right (-1.48 and -1.69 Gy) and the inferior directions (-0.94 and -1.03 Gy). For VMAT-5B, only isocenter shifts in the posterior, right, and inferior directions led to underdosage in CTV and the differences were in range of -0.79 ~ -0.15 Gy for ΔD_95%_ and -1.05 ~ -0.16 Gy for ΔD_98%_. For isocenter shifts in other directions, slight overdosage in CTV was observed (< 0.6 Gy). Dose inhomogeneity (ΔD_2%_ and ΔV_110%_) in both groups showed similar trends with ΔD_95%_ and ΔD_98%_. It’s interesting to note the values of ΔV_110%_ in VMAT-10B were very close to zero, indicating that setup errors had little effects on V_110%_ in VMAT-10B.

**Table 3:** Absolute differences in CTV between the perturbed and original plans.

Direction	ΔD_95%_ (Gy)		ΔD_98%_ (Gy)		ΔD_2%_ (Gy)		ΔV_110%_ (%)	
	VMAT-5B	VMAT-10B	VMAT-5B	VMAT-10B	VMAT-5B	VMAT-10B	VMAT-5B	VMAT-10B
A	0.50 ± 0.08	-0.25 ± 0.16	0.55 ± 0.11	-0.23 ± 0.16	0.64 ± 0.19	-0.07 ± 0.12	2.13 ± 2.33	0.02 ± 0.04
P	-0.79 ± 0.25	-1.69 ± 0.33	-1.05 ± 0.38	-1.99 ± 0.48	-0.59 ± 0.18	-0.85 ± 0.21	-0.43 ± 0.63	-0.02 ± 0.08
L	0.32 ± 0.09	-0.38 ± 0.18	0.37 ± 0.12	-0.40 ± 0.17	0.59 ± 0.19	-0.09 ± 0.19	1.87 ± 2.00	0.06 ± 0.12
R	-0.57 ± 0.19	-1.48 ± 0.23	-0.78 ± 0.32	-1.69 ± 0.35	-0.38 ± 0.13	-0.74 ± 0.20	-0.31 ± 0.46	-0.03 ± 0.08
S	0.19 ± 0.31	-0.70 ± 0.18	0.17 ± 0.30	-0.71 ± 0.15	0.19 ± 0.30	-0.41 ± 0.15	0.55 ± 1.00	-0.01 ± 0.08
I	-0.15 ± 0.07	-0.94 ± 0.14	-0.16 ± 0.07	-1.03 ± 0.14	-0.05 ± 0.10	-0.56 ± 0.17	-0.08 ± 0.28	-0.02 ± 0.08

A, anterior; P, posterior; L, left; R, right; S, superior; I, inferior.

**Fig 3:**
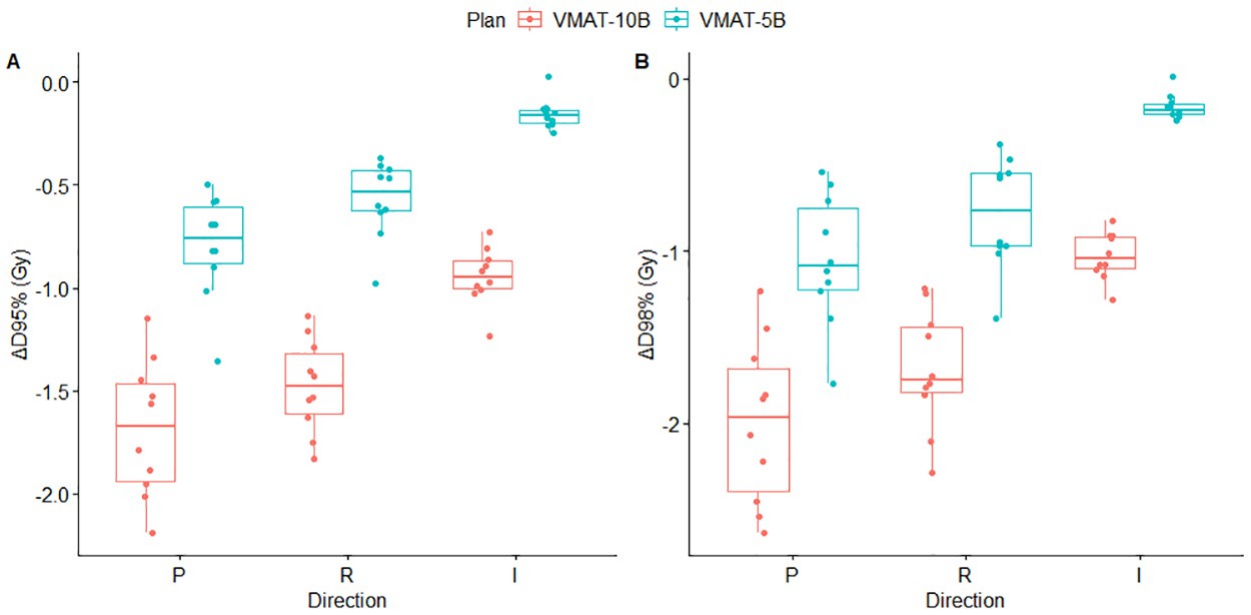
Box plot of ΔD_95%_ (A) and ΔD_98%_ (B) in VMAT-5B (green) and VMAT-10B (red) with perturbations in the posterior, right and inferior directions.

For better visualization of dose variations in CTV in VMAT-5B and VMAT-10B, boxplot of ΔD_95%_ and ΔD_98%_ against the posterior, right, and inferior directions was performed, and the results are presented in Fig 3. In line with the results in Table 3, the deviations in D_95%_ and D_98%_ in VMAT-5B were milder than that in VMAT-10B. Besides, for ΔD_95%_ and ΔD_98%_ in VMAT-10B, a large proportion of the plots were below -1.50 Gy (approximate -3.00% of the planned dose) in the posterior and right shifts. For VMAT-5B, almost all the plots were between-1.50 ~ 0.00 Gy in the three directions.

Dose fluctuations in the heart and left lung in VMAT-5B and VMAT-10B are shown in Tables 4 and 5. Only isocenter shifts in the posterior, right, and inferior directions increased dose to the organs, and the differences in VMAT-10B were smaller. For the heart, the average ΔD_mean_ were 0.42 Gy (range 0.10 ~ 0.95 Gy) and 0.20 Gy (range -0.11 ~ 0.72 Gy) in VMAT-5B and VMAT-10B, respectively. The ΔV_20Gy_ ranged from 0.18% to 2.19% and 0.00% to 1.97% for VMAT-5B and VMAT-10B, while the ΔV_30Gy_ ranged from 0.11% to 1.29% and 0.02% to 1.16% for VMAT-5B and VMAT-10B, respectively. The differences in the left lung were apparently larger than that in the heart. The average ΔD_mean_ were 1.20 Gy (range 0.79 ~ 1.18 Gy) in VMAT-5B and 0.68 Gy (range 0.47 ~ 0.84 Gy) in VMAT-10B. For ΔV_20Gy_, ΔV_30Gy_ and ΔV_40Gy_, the differences were in range of 1.61% to 2.86% in VMAT-5B and 1.17% to 2.45% in VMAT-10B. Posterior isocenter shifts contributed most to the dose fluctuations in the heart and the left lung as it involved more organ volumes in the treatment fields.

**Table 4:** Absolute differences in the heart between the perturbed and the original plans.

Direction	ΔV_20Gy_ (%)		ΔV_30Gy_ (%)		ΔD_mean_ (Gy)	
	VMAT-5B	VMAT-10B	VMAT-5B	VMAT-10B	VMAT-5B	VMAT-10B
A	-1.47 ± 0.68	-1.64 ± 0.69	-0.71 ± 0.48	-0.78 ± 0.51	-0.74 ± 0.22	-0.92 ± 0.22
P	2.19 ± 0.91	1.97 ± 0.85	1.29 ± 0.70	1.16 ± 0.65	0.95 ± 0.30	0.72 ± 0.28
L	-0.57 ± 0.18	-0.77 ± 0.19	-0.36 ± 0.21	-0.44 ± 0.25	-0.16 ± 0.07	-0.36 ± 0.09
R	0.72 ± 0.16	0.54 ± 0.16	0.46 ± 0.21	0.36 ± 0.19	0.21 ± 0.09	0.00 ± 0.09
S	-0.10 ± 0.16	-0.31 ± 0.13	-0.06 ± 0.09	-0.17 ± 0.10	-0.05 ± 0.07	-0.27 ± 0.07
I	0.18 ± 0.17	0.00 ± 0.19	0.11 ± 0.10	0.02 ± 0.11	0.10 ± 0.07	-0.11 ± 0.09

A, anterior; P, posterior; L, left; R, right; S, superior; I, inferior.

**Table 5:** Absolute differences in the left lung between the perturbed and the original plans.

Direction	ΔV_20Gy_ (%)		ΔV_30Gy_ (%)		ΔV_40Gy_ (%)		ΔD_mean_ (Gy)	
	VMAT-5B	VMAT-10B	VMAT-5B	VMAT-10B	VMAT-5B	VMAT-10B	VMAT-5B	VMAT-10B
A	-2.84 ± 0.35	-3.42 ± 0.47	-2.90 ± 0.35	-3.34 ± 0.41	-2.66 ± 0.36	-3.09 ± 0.43	-1.19 ± 0.15	-1.47 ± 0.19
P	2.71 ± 0.32	2.14 ± 0.28	2.86 ± 0.34	2.45 ± 0.29	2.78 ± 0.34	2.35 ± 0.28	1.18 ± 0.14	0.84 ± 0.11
L	-2.26 ± 0.32	-2.84 ± 0.35	-2.14 ± 0.27	-2.58 ± 0.28	-1.85 ± 0.25	-2.28 ± 0.29	-1.01 ± 0.13	-1.30 ± 0.15
R	2.40 ± 0.32	1.82 ± 0.37	2.31 ± 0.26	1.89 ± 0.25	2.08 ± 0.24	1.64 ± 0.21	1.08 ± 0.12	0.74 ± 0.12
S	-1.82 ± 0.30	-2.42 ± 0.44	-1.65 ± 0.26	-2.11 ± 0.31	-1.35 ± 0.22	-1.83 ± 0.23	-0.72 ± 0.10	-1.04 ± 0.12
I	1.91 ± 0.29	1.33 ± 0.23	1.82 ± 0.29	1.40 ± 0.27	1.61 ± 0.24	1.17 ± 0.23	0.79 ± 0.11	0.47 ± 0.11

A, anterior; P, posterior; L, left; R, right; S, superior; I, inferior.

## Discussion

VMAT has become common in PMRT of breast cancer, and 5 mm and 10 mm bolus are frequently selected to improve superficial target coverage [23, 24]. In this study, effects of bolus thickness on dose distribution and plan robustness of VMAT in left-sided breast cancer were investigated. We demonstrated that VMAT-10B had better dose coverage in CTV and homogeneity in PTV and CTV, whereas VMAT-5B was superior in OARs sparing (Tables 1 and 2). We also found that small setup uncertainties could induce dose deviations in CTV and OARs in VMAT plans. However, VMAT-5B appeared to be more robust than VMAT-10B because slight CTV underdosage and acceptable increased dose to the OARs were noted in this group.

Reportedly, couch shifts in patient setup were impossible to eliminate and the mean magnitudes were generally between 1 mm and 5 mm with imaging guidance [14–16]. Similar to previous data [14, 16], we found ~ 3 mm setup errors in all directions (unpublished) in a group of patients who underwent mastectomy and immediate implant-based reconstruction using weekly cone-beam CT (CBCT). Therefore, setup errors were simulated by shifting the isocenter of VMAT-5B and VMAT-10B by 3 mm along x, y and z axis. As expected, the errors led to dose fluctuations in CTV in both VMAT-5B and VMAT-10B. For VMAT-5B, dose disagreements in CTV D_95%_ and D_98%_ were small, with maximum variation of -1.05 Gy in D_98%_ when shifting the isocenter in the posterior direction (Table 3). The results were very close to those reported by Jensen et al. who evaluated plan robustness of VMAT to CBCT derived setup errors [29]. The authors attributed the limited dosimetric impacts of isocenter shifts to the robust optimization function in RayStation [29]. Unfortunately, the tool is not available for Varian’s Eclipse for VMAT. Moreover, only isocenter shifts in the posterior, right, and inferior directions compromised target coverage while setup errors in the other directions induced a slight rise in CTV D_95%_ and D_98%_. For VMAT-10B, the simulated setup errors led to underdosage in CTV in all directions. The ΔD_95%_ were between -1.69 and -0.25 Gy and the ΔD_98%_ were between -1.99 and -0.23 Gy (Table 3). Isocenter shifts in the posterior and right directions contributed most to the dose fluctuations. Using the same method, Liao et al. registered an average ΔD_95%_ of -0.6 Gy (range -1.40 ~ -0.10 Gy) and ΔD_98%_ of -1.0 Gy (range -2.80 ~ -0.30 Gy) when applying a 3 mm perturbation to the isocenter of VMAT with 10 mm bolus for left-sided breast cancer PMRT [19]. The data were in the same order of ours in this study. Fig 3 shows the distribution of ΔD_95%_ and ΔD_98%_ against perturbations in posterior, right and inferior directions in VMAT-5B and VMAT-10B. Clearly, most of the plots of VMAT-10B in the posterior and right directions were below -1.5 Gy, corresponding to approximate -3.00% of the planned dose, which were clinically unacceptable. For VMAT-5B, the differences were within -3.00% with shifts in all directions.

In contrast to our results, Liao et al. reported that setup errors in the anterior and left directions dramatically affected dose coverage in the targets [19]. We believe that the CTV-PTV margin should be the main reason for the difference. In this study, the PTV was obtained by uniformly adding a 5 mm margin. For plan evaluation, PTV was cropped at least 2 mm from skin surface and CTV was restricted to the edge of the PTV in the anterior direction. In the study by Liao et al., a smaller margin (3 mm) was employed to construct PTV. In addition, no description of cropping of PTV or CTV was found in their study [19]. Generally, isocenter shifts in the posterior and right correspond to move the target out of the region encompassed by PD, therefore, induces insufficient dose coverage in the target. For VMAT-10B, the extended isodose lines along the anterior chest wall were expected to account for the setup errors in the posterior and right directions. However, the dose fluctuations were pronounced in these directions. Recently, Oliver et al. evaluated skin dose resulting from chest wall irradiation by the means of Monte Carlo simulation using tangent and arc source model. They considered different bolus thicknesses and materials and found that 10 mm tissue-equivalent bolus could cause significant attenuation of the incident photons at near 55° by increasing the path length of the incident beams [27]. In this study, the oblique incident angle at near 55° has been registered for several arcs, as shown in Fig 1. We assume that the setup error may significantly change obliquity and/or path length of incident photons at near 55°, which in turn contributes to the pronounced underdosage in the target in VMAT-10B.

Lizondo et al. performed a systematic investigation to determine the optimal virtual bolus thickness and Hounsfield unit (HU) value for breast VMAT. The results showed that plans with 5 mm PTV extension, 10 mm virtual bolus and -400 HU were robust to 5 mm isocenter shifts in the breath direction (3.5mm along the x and y axis) based on relative differences in D_98%_ (< ±2.0%) and D_2%_ (< ±2.5%) [30]. In this work, the relative differences in D_98%_ and D_2%_ were up to -3.91% and -1.60%, respectively, in VMAT-10B with 3 mm posterior isocenter shifts. It can’t be concluded that VMAT with virtual bolus was more robust than VMAT-10B, since the targets for plan evaluation were cropped 5 mm inwards from the skin surface in the study of Lizondo et al. [30], whereas the targets around chest wall were only cropped 2 mm inside the body contour in our study. Factors including patient selection, beam arrangement and HU assignment to bolus could also lead to the differences between the studies.

**Fig 4:**
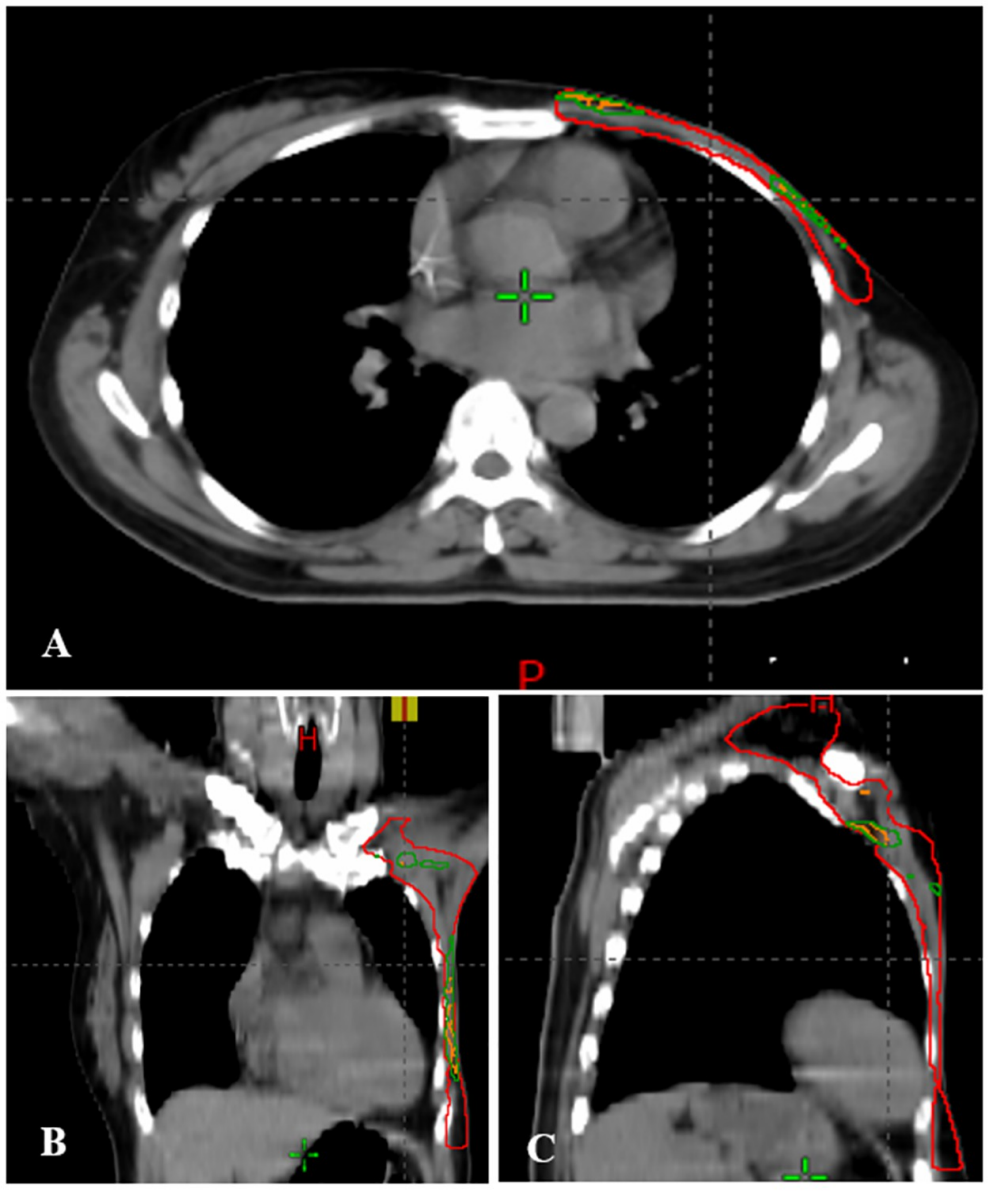
Underdosed volumes in CTV (red) in VMAT-5B (orange) and VMAT-10B (dark green) with setup errors in the posterior direction for a typical patient.

Liu et al. identified the association of local failures with setup uncertainties caused underdosage in head and neck cancer patients treated with VMAT. The underdosed volumes were either located at the edge or in the middle of the target [18]. Fig 4 presents the distribution of underdosed volumes in CTV of a typical patient in posteriorly perturbed VMAT-5B and VMAT-10B. Similarly, setup errors led to insufficient dose coverage not only at the edge but also in the middle of CTV along the chest wall or near the junction region. Moreover, the underdosed volumes in VMAT-5B primarily overlapped with those in VMAT-10B. This is reasonable because the VMAT-5B was recalculated from VMAT-10B without changing any parameters but bolus thickness. In line with the results in Table 3 and Fig 3, the underdosed volumes in perturbed VMAT-10B were significantly larger than that in perturbed VMAT-5B. For perturbations in the right and inferior directions, similar results were recorded and shown in S1 and S2 Figs.

It was suggested that dose differences in normal organs should be evaluated simultaneously with that of the target since a perturbation with little influence on target dose distribution might lead to overdose of the adjacent organs [19]. Dose disagreements in the heart and left lung were considered and estimated in this paper. Increased doses were observed with setup errors in the posterior, right, and inferior directions. The differences in the heart were generally milder than those in the left lung in VMAT-5B and VMAT-10B (Tables 4 and 5). The increments in VMAT-5B were higher than that in VMAT-10B. Similar to the changes in CTV, isocenter shifts in these directions may also affect obliquity and/or path length of incident beams in VMAT-10B, resulting in lower increments. Nevertheless, all the dosimetric parameters in the perturbed plans were still clinically acceptable, except the mean heart dose of four patients in VMAT-10B and one in VMAT-5B with isocenter shifts in the posterior direction (S2 File).

The improved long-term survival of breast cancer patients has encouraged radiation oncologists to fully consider the radiation-related toxicities, most notably heart toxicity. Radiation dose to the whole heart and volume of the organ receiving high dose was reported as risk factors of heart toxicity [31, 32]. According to Darby et al., the risk of major coronary events was linearly proportional to the mean heart dose by 7.4% per Gy and no clear threshold was observed [31]. Based on the paper, the estimated increase of ischemic heart disease is 7.03% and 5.33% for VMAT-5B and VMAT-10B with posterior perturbations, respectively. The results should be interpreted with caution because the absolute values in perturbed VMAT-5B remained slightly lower than those in perturbed VMAT-10B (S2 File). In another study based on randomized trials, the overall mean heart dose was 4.4 Gy and the estimated absolute risk of cardiac mortality was 0.3% for nonsmoking patients [33]. In this circumstance, the increased absolute risk for the perturbed plans could be very limited because the patient cohorts in our study were all nonsmokers and ΔD_mean_ for the heart was less than 1 Gy (Table 4).

The QUANTEC suggested V_25Gy_ < 10% to keep the probability of cardiac mortality within 1% in approximately fifteen years after radiation therapy [32]. Given that VMAT significantly reduces high dose volumes in the heart [9], a stricter dose constraint for the heart, V_20Gy_ < 10%, was utilized during plan optimization. As summarized in Table 2, the V_20Gy_ for the heart in VMAT-5B and VMAT-10B was around 3%. The parameter remained within 10% in the perturbed plans (S2 File), indicating the risk of cardiac mortality in our patient cohorts was acceptable.

Dosimetric advantages of multiple partial and split arcs over continuous long arcs were demonstrated in previous papers from our department [28] and other’s [34]. The mean heart dose reported by Lai et al. was 7.3 Gy [28], which was higher than that reported by Boman et al. (3.9 Gy for left-sided breast cancer with 240° split sub-arcs) [34] and those in this work (4.43 and 4.71 Gy for VMAT-5B and VMAT-10B, respectively). We assume that beam arrangements in the paper by Lai et al. may include more volume of the heart to the treatment fields, thus results in higher mean dose. Therefore, in this study, we improved the beam setting by using six partial arcs to separately cover the lymph nodes and chest wall (Fig 1). Collimator angles were carefully selected to minimize dose to the adjacent organs. The results from Boman et al. were slightly lower than ours because deep inspiration breath hold (DIBH) was used in several of their patients [34]. We also found that dose to the lungs was reduced in this study, compared with those in the paper by Lai et al. [28]. However, dose to the contralateral breast was slightly higher in our work, which may be attributed to the large field width of Arcs 1 and 2 that covered a part of the organ upon gantry rotation. Another pitfall of our six-arc VMAT was the treatment efficiency because monitor units were increased from 671 (range 619 ~ 695) to 1110 (range 965 ~ 1241) on average (S1 Table).

The main limitation of this study was the potential inaccuracy of the perturbed dose because it was directly recalculated based on the planning CT images without considering tissue deformations during the treatment course. Two recent studies from one center showed that optimizing breast VMAT with extended PTV and bolus resulted in higher robustness to tissue deformations than those without extension [35, 36]. According to Rossi et al., combination of 5 mm PTV extension and 8 mm optimization bolus was the best choice after exploring dose distribution in plans with various PTV extension (0, 5, and 7 mm) and optimization bolus (5, 8, and 10 mm) [35]. In the other study, the authors demonstrated that VMAT with 8 mm optimization bolus was able to account for up to 8 mm soft tissue deformations [36]. The plans in our study were optimized with 5 mm PTV extension and 10 mm bolus, which was close to the reported combination, thus the dosimetric effect of tissue deformations might be similar to that of the previous publications. To precisely assess dose fluctuations caused by setup errors, recalculation of plans in registered CBCT images is recommended.

Considering the factor that six-degree couch has not been universally used in the clinics, rotational errors are not discussed herein, which might be another limitation of our study. Furthermore, all the acquired data are based on the “one plan solution” for breast cancer, namely, bolus is used throughout the treatment course, which has been routinely used in our department [28] and others’ [19, 37]. For many other centers with two VMAT plans, one with bolus for a proportional of fractions and the other without bolus to reduce potential skin toxicity, the applicability of our results remains further confirmation. Finally, the sample size in this study was small. In order to obtain robust results, further investigations with more patients and detailed considerations are warranted.

## Conclusions

Small setup errors of 3 mm can cause dose fluctuations in CTV and adjacent organs including the heart and left lung in VMAT plans for PMRT of left-sided breast cancer. VMAT-5B results in acceptable dose reduction in CTV and increments in OARs when compared with VMAT-10B. Additionally, plans with 5 mm bolus deliver less dose to the OARs with acceptable target coverage and homogeneity. The 5 mm bolus is recommended for breast cancer PMRT with VMAT.

## Supporting information

S1 Fig Underdosed volumes in CTV (red) in VMAT-5B (orange) and VMAT-10B (dark green) with setup errors in right for a typical patient. (TIF)

S2 Fig Underdosed volumes in CTV (red) in VMAT-5B (orange) and VMAT-10B (dark green) with setup errors in the inferior direction for a typical patient. (TIF)

S1 File Dosimetry data of the targets and OARs for the patients with VMAT-5B and VMAT-10B. (XLSX)

S2 File Dosimetry of the targets and OARs for the patients with perturbed plans. (XLSX)

S1 Table Monitor units for the original plans in this study. (XLSX)
