# Supplementary figures and images for: Robustness of VMAT to setup errors in postmastectomy radiotherapy of left-sided breast cancer: Impact of bolus thickness
Source: PLoS One. 2023 Jan 24;18(1):e0280456. doi: 10.1371/journal.pone.0280456 (PMC9873183; doi:10.1371/journal.pone.0280456)

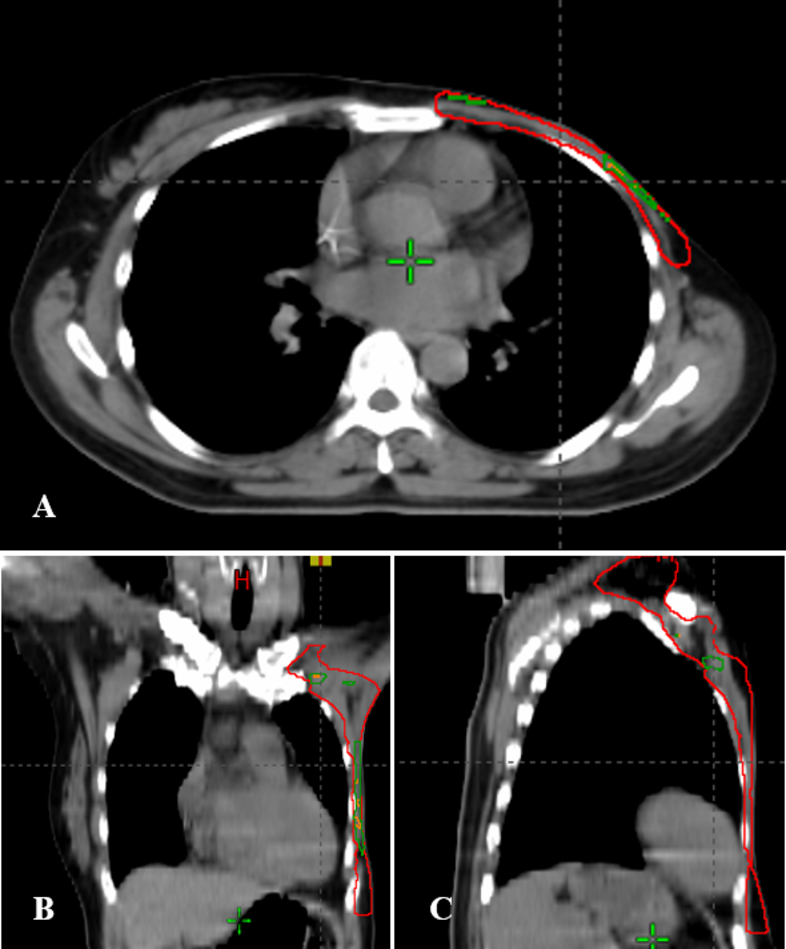

Supplement: S1 Fig — (TIF) [file pone.0280456.s001.tif]

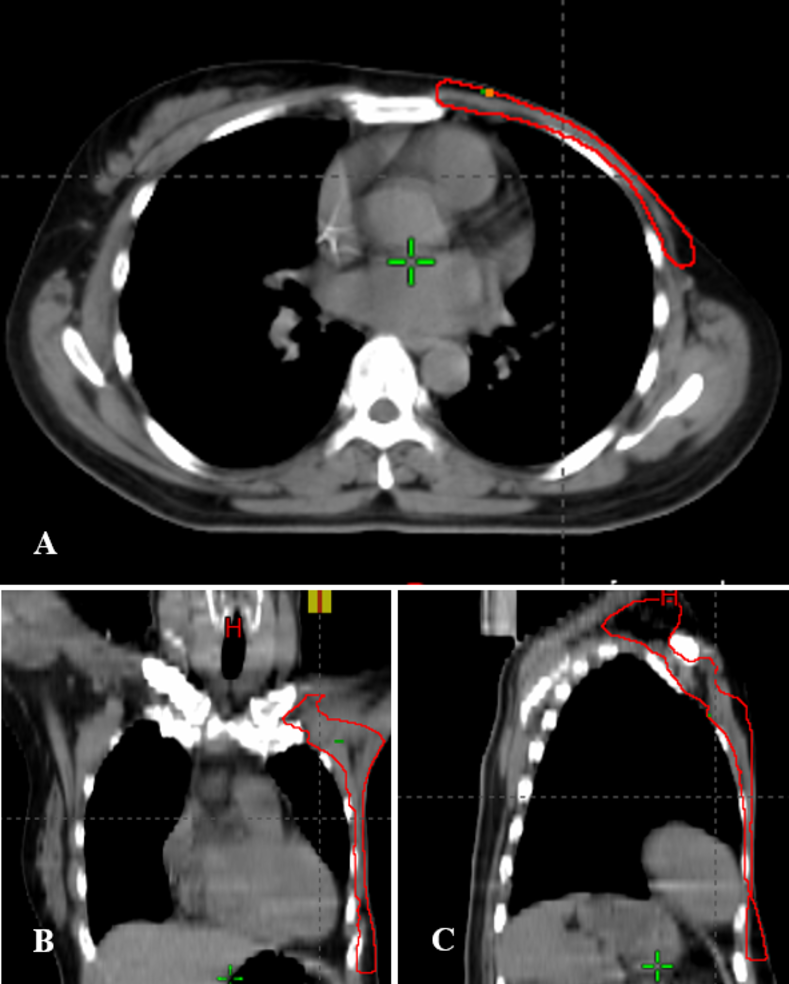

Supplement: S2 Fig — (TIF) [file pone.0280456.s002.tif]
